# Postoperative outcomes in surgical COVID-19 patients: a multicenter cohort study

**DOI:** 10.1186/s12871-021-01233-9

**Published:** 2021-01-12

**Authors:** François Martin Carrier, Éva Amzallag, Vincent Lecluyse, Geneviève Côté, Étienne J. Couture, Frédérick D’Aragon, Stanislas Kandelman, Alexis F. Turgeon, Alain Deschamps, Roy Nitulescu, Codjo Djignefa Djade, Martin Girard, Pierre Beaulieu, Philippe Richebé

**Affiliations:** 1 Department of Anesthesiology & Department of Medicine - Critical Care Division, Centre hospitalier de l’Université de Montréal, 1000, rue St-Denis, Porte D04-5028, Montréal, Québec H2X 3J4 Canada; 2 Carrefour de l’innovation et de l’évaluation en santé, Centre de recherche du CHUM, Montréal, Canada; 3 Department of Anesthesiology and Pain Medicine, Faculty of Medicine, Université de Montréal, Montréal, Canada; 4 Department of Anesthesiology, Hôpital du Sacré-Coeur de Montréal, CIUSSS du Nord de l’Île de Montréal, Montréal, Canada; 5 Department of Anesthesiology, Centre hospitalier Universitaire Sainte-Justine, Montréal, Canada; 6 Department of Anesthesiology and Critical Care Medicine, Division of Critical Care Medicine, Université Laval, Québec, Canada; 7 Department of Anesthesiology, Institut Universitaire de Cardiologie et de Pneumologie de Québec, Québec, Canada; 8 Department of Anesthesiology, Université de Sherbrooke, Sherbrooke, Canada; 9 Department of Anesthesiology, McGill University Health Center, Montréal, Canada; 10 Department of Anesthesiology & Department of Medicine, Population Health and Optimal Health Practices Research Unit (Trauma - Emergency - Critical Care Medicine), CHU de Québec - Université Laval Research Center, Québec, Canada; 11 Department of Anesthesiology, Institut de cardiologie de Montréal, Université de Montréal, Montréal, Canada; 12 Centre d’intégration et d’analyse des données médicales, Centre de recherche du CHUM, Montréal, Canada; 13 Imagerie et ingénierie, Centre de recherche du CHUM, Montréal, Canada; 14 Department of Anesthesiology, Centre hospitalier de l’Université de Montréal, Montréal, Canada; 15 Department of Anesthesiology, Hôpital Maisonneuve-Rosemont - CIUSSS de l’Est de l’île de Montréal, Montréal, Canada

**Keywords:** COVID-19, Surgery, Postoperative outcomes, Postoperative mortality, Health system impact, Pandemic

## Abstract

**Background:**

Data on postoperative outcomes of the COVID-19 patient population is limited. We described COVID-19 patients who underwent a surgery and the pandemic impact on surgical activities.

**Methods:**

We conducted a multicenter cohort study between March 13 and June 192,020. We included all COVID-19 patients who underwent surgery in nine centres of the Province of Québec, the Canadian province most afflicted by the pandemic. We also included concomitant suspected COVID-19 (subsequently confirmed not to have COVID-19) patients and patients who had recovered from it. We collected data on baseline characteristics, postoperative complications and postoperative mortality. Our primary outcome was 30-day mortality. We also collected data on overall surgical activities during this first wave and during the same period in 2019.

**Results:**

We included 44 COVID-19 patients, 18 suspected patients, and 18 patients who had recovered from COVID-19 at time of surgery. Among the 44 COVID-19 patients, 31 surgeries (71%) were urgent and 16 (36%) were major. In these patients, pulmonary complications were frequent (25%) and 30-day mortality was high (15.9%). This mortality was higher in patients with symptoms (23.1%) compared to those without symptoms (5.6%), although not statistically significant (*p* = 0.118). Of the total 22,616 cases performed among participating centres during the study period, only 0.19% had COVID-19 at the time of surgery. Fewer procedures were performed during the study period compared to the same period in 2019 (44,486 cases).

**Conclusion:**

In this Canadian cohort study, postoperative 30-day mortality in COVID-19 patients undergoing surgery was high (15.9%). Although few surgeries were performed on COVID-19 patients, the pandemic impact on surgical activity volume was important.

**Trial registration:**

ClinicalTrials.gov Identifier: NCT04458337.

**Supplementary Information:**

The online version contains supplementary material available at 10.1186/s12871-021-01233-9.

## Background

The world is experiencing a pandemic on a scale that has not been seen for many decades. Estimates of COVID-19 (Coronavirus Disease 2019) case fatalities are variable and have been reported to be between less than 1 and 7% [1–4]. Although resource utilization, such as hospitalization, and intensive care unit (ICU) admission, is well documented in COVID-19 patients, data on their surgical needs and outcomes remains limited [1–8].

Viral pneumonia is mostly a medical condition, but infected patients may require surgery [7–10]. To provide anesthetic and surgical care to COVID-19 patients, healthcare workers have to reorganize surgical platforms, personal protective equipment protocols, and in-hospital patient trajectories to prevent a viral spread to healthcare workers and other patients [11–15]. Documenting the needs for, and variety of, surgical procedures in this population is paramount in order to estimate the accrued risk for the patients.

Postoperative outcomes in SARS-CoV-2 infected patients have been previously reported [7, 8, 16]. Recently published data suggest an overall postoperative 30-day mortality between 19 and 24%, with more than half of the patients having postoperative pulmonary complications. These studies, mostly from Europe and the Middle East, did not report the overall impact of the pandemic on surgical activity. The surgical needs and postoperative outcomes of COVID-19 patients, as well as the overall access to surgical care during such a pandemic have yet to be fully evaluated in a Canadian setting.

To address this, we conducted a multicenter observational cohort study in the Province of Québec, the Canadian province most afflicted by the pandemic [17]. Our primary objective was to describe the perioperative characteristics of patients infected by SARS-CoV-2 who underwent surgery and their postoperative outcomes. Our secondary objectives were to explore the effect of the presence of symptoms on outcomes, describe the impact of the SARS-CoV-2 pandemic on overall surgical care and describe the characteristics and outcomes of suspected COVID-19 patients and those who had recovered from COVID-19 and underwent surgery during the same observation period. We hypothesized that the number of COVID-19 surgical patients would be small with a higher mortality among those with symptoms, that the impact of the pandemic on surgical care would be notable and that postoperative outcomes would be comparable between COVID-19 and suspected patients.

## Methods

### Design and setting

After Research Ethics Board approval from all centres, we conducted a multicenter observational cohort study based on a waived consent model in nine university hospitals in the Province of Québec from March 13, 2020, to June 19, 2020. This report follows STROBE guidelines for reporting observational studies [18].

### Study participants

We included all consecutive patients undergoing surgery who had tested positive for SARS-CoV-2 preoperatively. We defined positive SARS-CoV-2 infection by any positive Polymerase Chain Reaction (PCR) test (from either an oronasopharyngeal swab or an endotracheal aspirate) either before surgery or within 72 h after surgery and defined being symptomatic by the presence of any patient-reported COVID-19-related symptoms (fever, respiratory distress, etc).

We also included all patients undergoing surgery during the same period who were suspected of having COVID-19 at time of surgery (but subsequently tested negative) and all patients who had recovered from COVID-19. We defined suspected patients by the presence of the same COVID-19-related symptoms with unknown SARS-CoV-2 infection status at time of surgery (but later confirmed negative for SARS-CoV-2) which prompted the operating room team to take specific COVID-19-related precautions. We defined recovery as a patient who previously had a positive PCR test and then had two negative PCR tests for SARS-CoV-2 before surgery (or one negative test performed at least 14 days before surgery and the absence of symptoms at time of surgery). Patients were identified through the electronic medical data system or the operating room database specific to each site.

### Exposure variables

To address our primary objective, we first reported data on COVID-19 patients. To address our secondary objectives, we used the presence of COVID-19-related symptoms as an exposure variable and reported stratified data based on this variable for COVID-19 patients. To address another secondary objective, we used the COVID-19 disease status (confirmed, suspected, or recovered) as an exposure variable.

### Covariables

We collected baseline characteristics of the COVID-19 presentation such as the presence of symptoms at time of surgery, number of days since first symptoms or diagnosis, preoperative need for oxygen or invasive mechanical ventilation, and received treatments (e.g., antiviral agents, steroids, antibiotics). We also recorded demographic characteristics, baseline comorbidities, type of surgery, urgency of surgery, baseline laboratory values, preoperative Sequential Organ Failure Assessment (SOFA) score and intraoperative variables.

### Outcomes

Our primary outcome was 30-day survival after surgery. Our secondary outcomes were the postoperative occurrence of respiratory complications (atelectasis, pneumonia, Acute Respiratory Distress Syndrome [ARDS], and pulmonary aspiration), non-pulmonary infectious complications, acute kidney injury, thrombotic-associated complications (pulmonary embolism, myocardial infarction, stroke and cardiac arrest), hospital length of stay, 30-day mechanical ventilation-free days, 30-day organ dysfunction-free days, and any new ICU admission during the index admission. The endpoints we examined regarding the overall impact on surgical care were the number of cases performed during the inclusion period; the duration of the procedures; the required time to allow for operating room preparation, cleaning, and patient extubation; and other post-anesthesia care procedures. We collected similar data in all sites during a comparable period of observation (i.e., same dates) of the preceding year.

### Data measurement

We classified procedures into the following categories: neurosurgical (head and spine), cardiac, thoracic, major vascular (intrathoracic and/or intra-abdominal), non-vascular abdominal (laparotomy or laparoscopy), urogenital (urology and/or gynecology), non-spine orthopedic, peripheral vascular, otolaryngology-head-neck and other. Major surgery was defined as any neurosurgical, cardiac, thoracic, major vascular (cervical, intrathoracic and/or intra-abdominal) or non-vascular abdominal surgery (any intra-abdominal surgery). We also collected data on the surgical approach (minimally invasive, including laparoscopy, or invasive). Urgency of surgery was defined as the need to be performed within 24 h as requested by surgeons.

We measured survival at 30 days (within hospital or later after discharge if data available) and censored patients at last visit seen alive or at 30 days if still alive. We used existing definitions for postoperative pulmonary complications outcome (atelectasis, pneumonia, ARDS, pneumothorax) [19]. Non-pulmonary infectious complications were defined as any infection requiring antibiotics for > 72 h. Acute kidney injury was defined by the creatinine difference of the KDIGO-AKI criteria [20]. We defined thrombotic-related complications as reported by the treating physicians including pulmonary embolism, myocardial infarction, stroke or cardiac arrest. Mechanical ventilation was defined as the need for mechanical support (non-invasive or invasive). Organ dysfunction was defined as the need for vasopressors, mechanical ventilation and the use of renal replacement therapy after surgery [21]. The 30-day freedom from adverse outcomes included any day without the corresponding outcome.

### Data sources and management

Data was either collected prospectively by the clinical and the research team or retrospectively collected the days following surgery. Data was entered at each site into a centralized electronic database following a manual of standard operating procedures and individual site training. Outcomes were adjudicated by a physician at each site. The surgical impact data was extracted as aggregate data from operating room administrative systems of each centre.

### Statistical analyses

We included a convenience sample of all eligible patients who underwent surgery during the observation period. Due to the limited sample size, the reported analyses are primarily descriptive. We reported continuous variables as mean (standard deviations (SD)) or median [interquartile range [IQR]] for skewed distributions) and categorical variables as proportions. We reported 30-day survival using Kaplan-Meier estimates with 95% confidence intervals (CI) stratified by subgroups. To compare symptomatic COVID-19 patients to asymptomatic ones, and COVID-19 patients to suspected ones and those who had recovered, we performed log-rank tests. Finally, we conducted a post hoc sensitivity analysis by removing patients for whom a tracheotomy was the surgical procedure performed, since they may have a different postoperative trajectory. We set our alpha level at 0.05. All analyses were performed with R statistical software (R Core Team, version 4.0.2).

## Results

Baseline and surgical characteristics are reported in Table 1 for COVID-19 patients and in Table S2 for other patients, while surgical specialties are reported in Table S1 for all included patients.

**Table 1:** COVID-19 patients characteristics

Variables	COVID-19 patients (***n*** = 44)	Symptomatic (***n*** = 26)	Asymptomatic (***n*** = 18)
**Demographics**			
Age (years)	59 (22)	61 (24)	58 (20)
Sex (female)	24 (55%)	13 (50%)	11 (61%)
**Comorbidities**			
BMI (kg·m^−2^)^a^	30 (7)	29 (5)	32 (9)
Diabetes	11 (25%)	7 (27%)	4 (22%)
Hypertension	23 (52%)	13 (50%)	10 (56%)
**COVID-19 symptoms and treatment at surgery**			
Positive test at surgery	42 (96%)	24 (92%)	18 (100%)
Days since positive test (days)	5 [2, 19]	8 [2, 20]	3 [1, 16]
Cough	17 (39%)	17 (65%)	0 (0%)
Dyspnea	13 (30%)	13 (50%)	0 (0%)
Respiratory distress	9 (21%)	9 (35%)	0 (0%)
Fever	17 (39%)	17 (65%)	0 (0%)
Antibiotics	24 (55%)	19 (73%)	5 (28%)
Steroids	11 (25%)	9 (35%)	2 (11%)
**Preoperative respiratory and organ dysfunction**			
Oxygen	12 (27%)	11 (42%)	1 (6%)
Mechanical ventilation	8 (18%)	8 (31%)	0 (0%)
SOFA score^b^	0 [0, 3]	1 [0, 6]	0 [0, 1]
**Surgical characteristics**			
General anesthesia	28 (64%)	18 (69%)	10 (56%)
Urgency^c^	31 (71%)	19 (73%)	12 (67%)
Major surgery	16 (36%)	9 (35%)	7 (39%)
Blood loss^d^ (mL)	175 [75, 500]	150 [100, 600]	250 [50, 300]

Data is reported as mean (SD), as median [q1, q3] or as number of events (proportion in %)

N.B. SOFA is not reported because of excessive missing values

*Abbreviations*: *BMI* Body mass index, *SOFA* Sequential Organ Function Assessment score

^a^ 22 missing values (12 in symptomatic and 8 in asymptomatic patients)

^b^ When no bilirubin was measured preoperatively, we imputated a value of 0 for the liver component of the SOFA score. 1 missing value in each subgroup

^c^ Urgency was defined by the need to undergo a surgery within 24 h

^d^ 12 missing values (8 in symptomatic and 4 in asymptomatic patients)

### COVID-19 patients

**Fig. 1:**
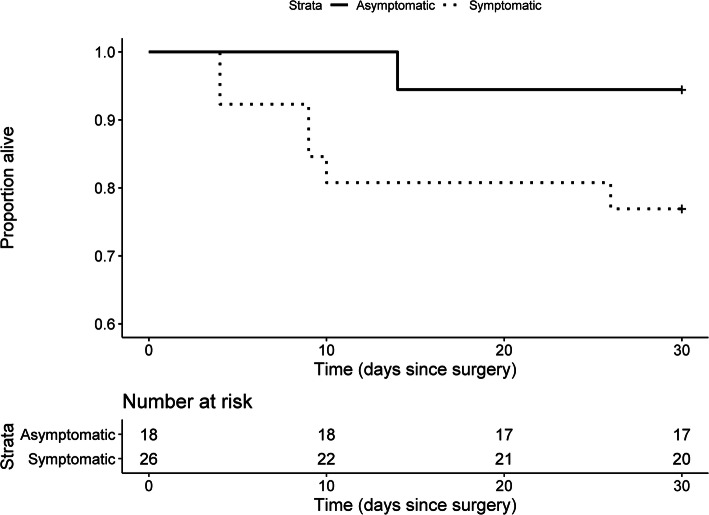
Kaplan-Meir curves for 30 -day postoperative survival in COVID-19 patients. *P = 0.118 by log-rank test*

Forty-four patients (42 with a preoperative positive PCR test) with COVID-19 underwent a surgical procedure between March 13, 2020, and June 19, 2020. Among these 44 patients with COVID-19, 26 patients were symptomatic at time of surgery with 18 being asymptomatic carriers. Demographics and surgical characteristics were similar between these groups, except for preoperative treatments and respiratory support (Table 1). In these patients, 71% of surgeries were urgent, 36% were major ones and 64% were performed under general anesthesia; these characteristics seemed to be similar between symptomatic and asymptomatic patients (Table 1). Complications were relatively rare, with the exception of pulmonary complications (25%) and new ICU admissions (27%). These complications seemed to be higher in symptomatic COVID-19 patients (Table 2). The overall 30-day mortality was 15.9% in these patients (Table 2). This mortality was numerically higher in symptomatic patients (23.1% in symptomatic patients and 5.6% in asymptomatic patients), although the observed difference between strata was not significant (*P* = 0.12) (Table 2 and Fig. 1). As a sensitivity analysis, we excluded six COVID-19 patients who had a tracheotomy as a surgical procedure. In this subgroup of 38 patients, 2 were under invasive mechanical ventilation at surgery and 5 died within 30 days after surgery (13.2%) (not shown in tables).

**Table 2:** Patients complications up to 30 days after surgery

Variables	COVID-19 patients (***n*** = 44)	Symptomatic (***n*** = 26)	Asymptomatic (***n*** = 18)
**Complications**			
Pulmonary complications	11 (25%)	9 (35%)	2 (11%)
Infectious complications (non-pulmonary)	4 (9%)	1 (4%)	3 (17%)
Acute kidney injury^a^	8 (18%)	4 (15%)	4 (22%)
Thromboembolic complications	2 (5%)	2 (8%)	0 (0%)
**Resource utilization**			
New ICU admissions	12 (27%)	10 (39%)	2 (11%)
Hospital length of stay	17 [4, 36]	22 [6, 40]	7 [3, 22]
Mechanical ventilation free days (at 30 days)	27.0 (7.4)	25.0 (9.1)	29.9 (0.3)
Organ dysfunction-free days (at 30 days)	25.2 (9.3)	22.2 (11.1)	29.6 (1.4)
**Mortality**			
30-day mortality	7 (15.9%)	6 (23.1%)	1 (5.6%)
Kaplan-Meir survival probability^b^	0.84 [0.74, 0.96]	0.77 [0.62, 0.95]	0.94 [0.84, 1.00]

Data is reported as mean (SD), as median [q1, q3] or as number of events (proportion in %)

*Abbreviations*: *ICU* Intensive care unit

^a^ No acute kidney injury required renal replacement therapy

^b^ 30-day survival probability from the estimated Kaplan-Meir survival function, expressed with 95% confidence intervals

### Impact on surgical care

During this 3-month long first wave of the pandemic, the total number of surgical procedures decreased by 50% as compared to the same time period in 2019 (22,616 cases in 2020 compared to 44,486 cases in 2019; Table 3). Of these 22,616 surgical cases, only 44 (0.19%) had COVID-19.

**Table 3:** Number of surgical procedures and operating room utilization at each centre

Center	COVID-19 cases	Total number of surgical procedures^**a**^	% of COVID-19 cases	Mean time from OR entry to exit per case^**b**^	PACU care in the OR^**c**^	Total number of surgical procedures	Mean time from OR entry to exit^**b**^
	March 13 to June 19, 2020					March 13 to June 19, 2019	
1	8	3960	0.20%	02:43	Yes^d^	7460	02:04
2	10	3153	0.32%	01:33	Yes	6586	01:24
3	1	2385	0.04%	NA	No	2590	NA
4	18	947	1.90%	02:53	Yes	2471	02:16
5	2	NA	NA	NA	NA	NA	NA
6	0	601	NA	04:30	Yes	540	04:19
7	1	2637	0.04%	02:45	No	5555	02:11
8	2	7948	0.03%	01:36	Yes	17,847	01:54
9	2	985	0.20%	03:23	Yes	1437	03:16
TOTAL	44	22,616	0.19%^e^	2:11^f^	–	44,486	1:59^e^

N.B. Cleaning and preparation data was missing for most centres. Data was produced from operating room administrative systems, thereby precluding producing any dispersion statistics

*Abbreviations*: *OR* Operating room, *PACU* Post-Anesthesia Care Unit, *NA* Not available

^a^ Number of surgical cases includes all patients who underwent a surgical intervention, including patients with a laboratory confirmed SARS-CoV-2 infection

^b^ Expressed as hours:minutes

^c^ PACU care provided in the OR for all COVID-19 confirmed, suspected or high-risk patients

^d^ PACU care provided in the OR for all COVID-19 confirmed or suspected and during the first 25 min after extubation for high-risk patients

^e^ The 2 patients from centre #5 were excluded from the numerator

^f^ Weighted mean

### COVID-19 suspected patients and patients who had recovered from COVID-19

We included 18 suspected patients and 18 patients who had recovered from COVID-19 (Tables S2 and S3). Suspected patients had an incidence of pulmonary complications and new ICU admissions close to symptomatic COVID-19 patients but seemed to have a slightly lower 30-day mortality (16.7% versus 23.1%) (Table S3). One suspected patient suffered from a cardiac arrest and survived. Patients who had recovered from COVID-19 seemed to be comparable to asymptomatic patients regarding their complications profile (Tables S2 and S3). The observed difference in survival between COVID-19, suspected and patients who had recovered was not statistically significant (*P* = 0.55, Figure S1).

## Discussion

This study provides data on surgeries performed in COVID-19 patients and their postoperative outcomes in the Canadian province most afflicted by the pandemic [17]. We observed an important postoperative 30-day mortality of 15.9% in patients undergoing surgery with COVID-19, potentially from symptomatic patients although we could not conclude. We observed a 50% relative reduction in overall surgical activities during the pandemic in most university hospitals of the province of Quebec.

Overall, SARS-CoV-2 infected patients have not experienced many surgeries during this pandemic wave (< 0.2% of surgical cases). This observation is probably a combination of limited surgical needs in this population and a restriction to surgical care imposed on them until they recover from their infection to potentially reduce postoperative complications [22, 23]. As such, they required mostly urgent minor surgery, although 36% of them required a major one. Routine preoperative testing for SARS-CoV-2 itself has been recently associated with less postoperative pulmonary complications in major surgeries, probably by allowing to postpone or cancel surgery in active cases [24]. Such intervention was not routinely applied to all surgical patients in the Province of Quebec during the first wave due to limited supply of reactants. Nonetheless, 25% of the patients suffered from a pulmonary complications, which is lower than other reported incidences [7, 8]. Similarly to another cohort of COVID-19 surgical patients, we observed a small incidence of thromboembolic complications (4.5%) for a population of patients undergoing mostly urgent surgeries [7].

Our observed 30-day mortality of 15.9% seemed to be lower than the 19.5 to 23.9% reported mortalities in other cohort studies [7, 8, 16]. The possible discrepancy might be explained by random variation, different patient selection for surgery or different overall perioperative care. However, our Kaplan-Meir 30-day survival probability confidence interval in COVID-19 patients was wide (from 0.74 to 0.96), suggesting that our observation was likely to not be significantly different from other ones. One group compared postoperative outcomes in 41 COVID-19 patients to 82 non-COVID-19 patients matched by surgical disease [8]. They observed a higher proportion of complications and mortality in COVID-19 patients, but non-COVID-19 patients had a better preoperative respiratory function, did not need any mechanical ventilation and were not in septic shock at time of surgery [7, 8]. We observed a relatively comparable mortality between suspected patients (16.7%) and symptomatic COVID-19 patients (23.1%), suggesting that postoperative mortality may be secondary to the presence of an active infectious process at surgery. Such observation could also be explained by false negative COVID-19 results in suspected patients, potentially aligning their outcome to those of COVID-19 patients [25]. Overall, like other groups, we observed a moderately high 30-day postoperative mortality that was higher than expected in non-cardiac surgery (1–4%) [26, 27].

Surgical care should not be overlooked during a pandemic. Even though mobilizing surgical ward and operating room resources to care for SARS-CoV-2 infected patients, population’s surgical needs have to be fulfilled [15, 28, 29]. Compared to the previous year, more than 22,000 patients over 9 hospitals in the province of Quebec did not receive timely surgical care during our period of observation. In one centre (centre #1), 8 out of 420 patients hospitalized for COVID-19 needed surgery (data not reported previously), while 3500 surgical cases were cancelled during this period. In the greater Toronto area hospitals, all surgical activities were reduced by as much as 37 to 70% for both inpatient and outpatient surgeries during the SARS pandemic in April 2003 [30]. During the first wave of the COVID-19 pandemic, we observed a reduction in surgical activity of 50% over 3 months in 9 participating centres, although the impact is probably beyond what we observed. Thus, a significant backlog of surgeries will have to be undertaken while the health system is still stressed by the pandemic [23]. Real-time system utilization indicators should be further developed and applied during a pandemic to help adapt surgical elective programs within system overwhelming prevention strategies as well as “COVID-19 free pathways” to prevent cross-contamination [31, 32]. Allocating care to COVID-19 patients is paramount, but this should not be done at the price of over restricting care in surgical patients to ensure proportional resource allocation across all population subgroups.

Our study has limitations. Its main limitation is the small sample size. While this highlights the limited number of surgeries performed in this population, it precluded any regression-based quantitative analysis on determinants of poor postoperative outcomes. We did not have the power to quantitatively measure association with either the presence of symptoms in COVID-19 or the presence of an active infection compared to our controls. Also, our comparators are biased, since “suspected” patients were by definition sick patients and “patients who had recovered” were fit enough patients to undergo an elective surgical procedure. Since we included patients tested positive for SARS-CoV-2, we could have missed untested patients, especially asymptomatic ones. However, this was probably limited since all patients having compatible symptoms or any risk factor for SARS-CoV-2 based on Québec Public Health Agency guidelines were tested prior to surgery. Our observations must therefore be interpreted as descriptive and exploratory. On the other hand, we included patients from many centers, providing relative generalizable results. Despite these limitations, we were able to draw a perspective of the surgical care during the current pandemic in Québec, Canada’s hardest hit province with almost half of the national COVID-19 cases.

## Conclusions

Our findings from this multicenter Canadian cohort study suggest that COVID-19 patients undergoing a surgery have a relatively high postoperative mortality. Our results also suggest that the pandemic had important effects on the overall conduct of surgical care despite limited utilization of surgical resources by COVID-19 patients.

## Supplementary Information

**Additional file 1.**

## Data Availability

The datasets generated and analyzed during the current study are not publicly available due to legal restrictions but are available from the corresponding author on reasonable written request and local REB approval. The Province of Quebec does not allow public patient data sharing. The dataset is held on a secured server at the CHUM Research Center.

## Abbreviations

SARS-CoV-2: Severe Acute Respiratory Syndrome Coronavirus type 2
COVID-19: Coronavirus Disease 2019
ICU: Intensive care unit
PCR: Polymerase Chain Reaction
SOFA: Sequential Organ Failure Assessment
KDIGO-AKI: Kidney Diseases Improving Global Outcomes Acute Kidney Injury
