# Supplementary material for: Postoperative outcomes in surgical COVID-19 patients: a multicenter cohort study
Source: BMC Anesthesiol. 2021 Jan 12;21:15. doi: 10.1186/s12871-021-01233-9 (PMC7801565; doi:10.1186/s12871-021-01233-9)
Supplement: Supplementary file 1 — Additional file 1. [file 12871_2021_1233_MOESM1_ESM.pdf]

Supplementary material

Postoperative outcomes in surgical COVID-19 patients – a multicenter cohort study

François M. Carrier, MD MSc[1,2,3]
Éva Amzallag, MSc[2]
Vincent Lecluyse, MD[3,4]
Geneviève Côté, MD MSc[3,5]
Etienne J. Couture, MD[6,7]
Frédérick D'Aragon, MD MSc[8]
Stanislas Kandelman, MD[9]
Alexis F. Turgeon, MD MSc[6,10]
Alain Deschamps, MD PhD[3,11]
Roy Nitulescu, MA[12]
Codjo Djignefa Djade, MSc[12]
Martin Girard, MD[1,3,13,14]
Pierre Beaulieu, MD PhD[3,14]
Philippe Richebé, MD PhD[3,15]

[1] Department of Anesthesiology & Department of Medicine - Critical Care Division, Centre hospitalier de l'Université de Montréal
[2] Carrefour de l'innovation et de l'évaluation en santé, Centre de recherche du CHUM
[3] Department of Anesthesiology and Pain Medicine, Faculty of Medicine, Université de Montréal
[4] Department of Anesthesiology, Hôpital du Sacré-Coeur de Montréal, CIUSSS du Nord de l'Île de Montréal
[5] Department of Anesthesiology, Centre hospitalier Universitaire Sainte-Justine
[6] Department of Anesthesiology and Critical Care Medicine, Division of Critical Care Medicine, Université Laval
[7] Institut universitaire de cardiologie et de pneumologie de Québec
[8] Department of Anesthesiology, Université de Sherbrooke
[9] Department of Anesthesiology, McGill University Health Center
[10] Department of Anesthesiology & Department of Medicine – CHU de Québec - Université Laval Research Center, Population Health and Optimal Health Practices Research Unit (Trauma - Emergency - Critical Care Medicine)
[11] Department of Anesthesiology, Institut de cardiologie de Montréal, Université de Montréal
[12] Centre d'intégration et d'analyse des données médicales, Centre de recherche du CHUM
[13] Imagerie et ingénierie, Centre de recherche du CHUM
[14] Department of Anesthesiology, Centre hospitalier de l'Université de Montréal
[15] Department of Anesthesiology and Pain Medicine, Hôpital Maisonneuve-Rosemont, CIUSSS de l'Est de l'île de Montréal

Table S1. Surgical specialties

Variables	Category	Symptomatic (n=26)	Asymptomatic (n=18)	Suspected (n=18)	Recovered (n=18)
Type of surgery	Major	9 (35%)	7 (39%)	5 (28%)	4 (22%)
	Minor	17 (65%)	11 (61%)	13 (72%)	14 (78%)
Type of major surgical specialty	Abdominal	3 (12%)	2 (11%)	4 (22%)	2 (11%)
	Neurosurgery	5 (19%)	5 (28%)	0 (0%)	1 (6%)
	Thoracic	0 (0%)	0 (0%)	1 (6%)	1 (6%)
	Vascular	1 (4%)	0 (0%)	0 (0%)	0 (0%)
Type of minor surgical specialty	ENT	6 (23%)	0 (0%)	2 (11%)	3 (17%)
	Obstetrics	4 (15%)	4 (22%)	1 (6%)	0 (0%)
	Orthopedic (excl. spinal)	2 (8%)	4 (22%)	4 (22%)	4 (22%)
	Other	0 (0%)	0 (0%)	0 (0%)	1 (6%)
	Peripheral vascular	2 (8%)	0 (0%)	0 (0%)	0 (0%)
	Plastic surgery	0 (0%)	1 (6%)	2 (11%)	2 (11%)
	Urogenital	3 (12%)	2 (11%)	4 (22%)	4 (22%)

Table S2. Overall patients' characteristics

Variables	Suspected (n = 18)	Recovered (n = 18)
**Demographics**		
Age	51.4 (28.5)	60.7 (17.6)
Sex (female)	8 (44%)	10 (56%)
**Comorbidities**		
Diabetes	3 (17%)	5 (28%)
Hypertension	7 (39%)	9 (50%)
**COVID-19 symptoms and treatment at surgery**		
Days since positive test or clinical diagnosis[1]	1 [0, 1]	23 [43, 46]
Cough	4 (22%)	1 (6%)
Dyspnea	3 (17%)	2 (11%)
Respiratory distress	2 (11%)	1 (6%)
Fever	7 (39%)	2 (11%)
Antibiotics	8 (44%)	3 (17%)
Steroids	5 (28%)	0 (0%)
**Preoperative respiratory and organ dysfunction**		
Oxygen	7 (39%)	2 (11%)
Invasive respiratory support	3 (17%)	2 (11%)
SOFA score[2]	3 [0, 7]	1 [0, 2]
**Surgical characteristics**		
General anesthesia	13 (72%)	11 (61%)
Urgency[3]	15 (83%)	6 (33%)
Major surgery	5 (28%)	4 (22%)
Blood loss[4]	225 [150, 475]	300 [100, 1500]

Data is reported as mean (SD), as median [q1, q3] or as number of events (proportion in %).
[1] Positive test for patients who had recovered and clinical suspected diagnosis for suspected patients.
[2] When no bilirubin was measured preoperatively, we imputated a value of 0 for the liver component of the SOFA score. 2 missing values in "suspected" and 1 missing value in "who had recovered" subgroup.
[3] Urgency was defined by the need to undergo a surgery within 24 hours.
[4] 10 missing values in suspected patients and 8 in patients who had recovered.
N.B. BMI is not reported because of excessive missing values.
Abbreviations: BMI = body mass index, SOFA = Sequential Organ Function Assessment score

Table S3. Patients' complications up to 30 days after surgery

Variables	Suspected (n = 18)	Recovered (n = 18)
**Complications**		
Pulmonary complications	6 (33%)	1 (6%)
Infectious complications (non-pulmonary)	3 (17%)	2 (11%)
AKI[1]	5 (28%)	0 (0%)
Thromboembolic complications	3 (17%)	0 (0%)
**Resource utilization**		
New ICU admissions	9 (50%)	5 (28%)
Hospital length of stay	10 [3, 22]	3 [0, 16]
Mechanical ventilation free days (at 30 days)	27.0 (4.1)	29.3 (1.6)
Organ dysfunction free days (at 30 days)	20.4 (11.5)	28.7 (3.9)
**Mortality**		
30-day mortality	3 (16.7%)	1 (5.6%)
Kaplan-Meir survival probability[2]	0.83 [0.68, 1.00]	0.94 [0.84, 1.00]

Data is reported as mean (SD), as median [q1, q3] or as number of events (proportion in %).

[1] 3 patients required renal replacement therapy in the suspected group.

[2] 30-day survival probability from the estimated Kaplan-Meir survival function, expressed with 95% confidence intervals.

Abbreviations: AKI = acute kidney injury, ICU = intensive care unit

Figure S1. Kaplan-Meier curves of COVID-19, suspected and recovered patients.

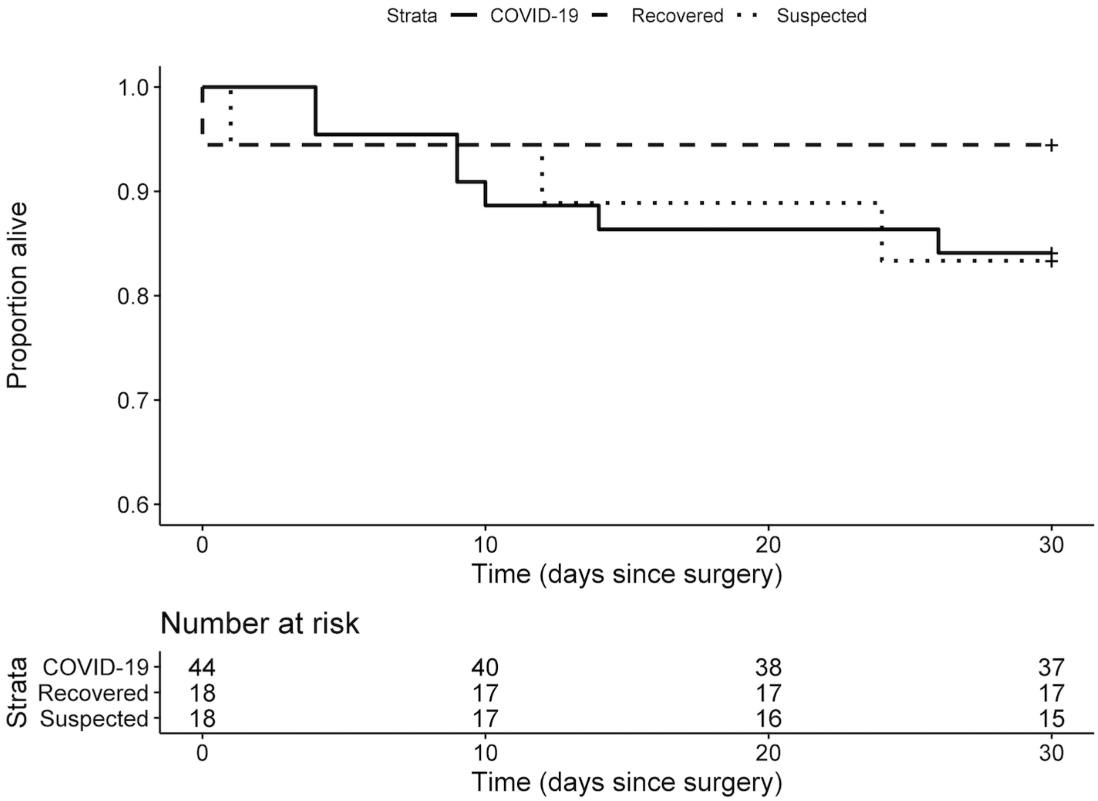

P = 0.554 by log-rank test

List of participating centres:

- Centre hospitalier de l'Université de Montréal
- Centre hospitalier Universitaire Sainte-Justine
- Hôpital du Sacré-Cœur-de-Montréal, CIUSSS du Nord de l'Île de Montréal
- Institut de Cardiologie de Montréal
- Hôpital Maisonneuve-Rosemont, CIUSSS de l'Est de l'île de Montréal
- McGill University Health Center
- Centre hospitalier Universitaire de Sherbrooke
- CHU de Québec – Université Laval
- Institut universitaire de cardiologie et de pneumologie de Québec - Université Laval
